# Museum-based art activities to stay young at heart? Results of a randomized controlled trial

**DOI:** 10.3389/fmed.2023.1184040

**Published:** 2024-01-05

**Authors:** Margot Cami, Océane Planta, Jacqueline Matskiv, Alexandra Plonka, Auriane Gros, Olivier Beauchet

**Affiliations:** ^1^Research Centre of the Geriatric University Institute of Montreal, Montreal, QC, Canada; ^2^Université Côté d'Azur, Centre Hospitalier Universitaire de Nice, Laboratoire CoBTeK, Service Clinique Gériatrique du Cerveau et du Mouvement, Nice, France; ^3^Department of Medicine and Geriatrics, University of Montreal, Montreal, QC, Canada; ^4^Department of Medicine, Division of Geriatric Medicine, Sir Mortimer B. Davis Jewish General Hospital and Lady Davis Institute for Medical Research, McGill University, Montreal, QC, Canada

**Keywords:** art, health, museum, older adult, clinical trial

## Abstract

**Background:**

Health benefits have been reported with art activities. Heart rate is a biomarker of health state. The aim of this randomized controlled trial (RCT) was to compare the changes in heart rate over a 3 month-period in older adults participating in art-based activities at the Montreal Museum of Fine Arts (MMFA, Quebec, Canada) and in their control counterparts.

**Methods/design:**

Participants (mean age 71.0 ± 5.1; 84.9% female) were a subset of older community dwellers recruited in a RCT in two parallel groups (*n* = 28 in the intervention group and *n* = 25 in the control group) who had their heart rate recorded. They attended weekly participatory MMFA-based art activities over a 3-month period. Heart rate was collected via the smart watch Fitbit Alta HR at baseline (M0) and at 3 months (M3). The outcomes were mean heart rate per hour for the full day, including active and inactive hours.

**Results:**

Heart rate for full day (*p* = 0.018) and active hours (*p* = 0.028) were slower in the intervention group compared to the control group. Decrease in mean heart rate for full day between M0 and M3 in the intervention group was higher than in the control group (*p* = 0.030). The linear regression showed that MMFA-based art activities decreased full day heart rate (Coefficient of regression Beta = −6.2 with *p* = 0.010).

**Conclusion:**

MMFA-based art activities significantly decreased full day heart rate, suggesting a health benefit in older community dwellers who participated in the RCT.

**Clinical trial registration**: NCT03679715.

## 1 Introduction

Museum-based activities like guided visits or art-making workshops demonstrate potential to improve the mental and physical health of older adults (1–5). Most existing studies that attest to the health benefits of museum-based arts activities use self-reported questionnaires for the health assessment (2–8). The main disadvantage of these questionnaires is the subjectivity of responses, which may result in inaccurate or invalid answers (9). In addition, today’s questioning is not whether art has benefits on health but what are the mechanisms of these benefits. Few studies explored this avenue but showed a positive association between proxy measures for the experience of stress in humans and visual art (5). For instance, art making resulted in significant lowering of cortisol levels salivary (10). Thus, examining health benefits of art requires to employ objective measures. To date, there is no information about museum-based activities effect on heart rate, which is a proxy measure of autonomic nervous system functioning (11).

A healthy state requires normal physiological functions, which are regulated in part through the autonomic nervous system, itself composed of the sympathetic and the parasympathetic nervous systems (12–14). Together, these two systems control vegetative functions and the stress response (14–16). Heart rate is controlled by the autonomic nervous system (12). The sympathetic and parasympathetic nervous systems increase and suppress heart rate, respectively (12–16). Heart rate is a simple, non-invasive, and objective measure that is easily recordable in daily living activities via smart watches (17). Aging is often associated with an imbalance in autonomic nervous system activity, with sympathetic activity increasing in comparison to parasympathetic activity, ultimately leading to an increased heart rate (13–15). This imbalance results in an inability to control stress response that, over time, may lead to an unhealthy state (14, 18). For instance, higher resting heart rate was associated with worse functional status and with higher risk of future functional decline in older adults, independent of cardiovascular diseases (19). It has been showed that heart rate may improve with mindfulness practice (20). There are few data about the effects of museum-based activities on physiological measures depending on sympathetic and parasympathetic nervous system balance like heart rate and blood pressure. It has been reported in a clinical trial that participants who were exposed to figurative art significantly decreased their systolic blood pressure compared to those exposed to modern art and museum office, but no effects were observed in the heart rate (21).

Recently we performed a randomized controlled trial (RCT) with the aim to compare changes in frailty status, well-being and quality of life in community-dwelling older adults living in Montreal (Quebec, Canada) participating in a 3-month session of weekly “Thursdays at the Museum” carried out at the Montreal Museum of Fine Arts (MMFA) and in their control counterparts who did not participate in MMFA-based art acitivities (8). A total of 165 older community dwellers were recruited in this RCT with two parallel groups (intervention vs. control). The intervention group showed significant improvements to frailty, well-being and quality of life when compared to the control group. This health benefit suggests that sympathetic and parasympathetic nervous system activities were more balanced in the intervention group, suggesting in a lower heart rate in the intervention group compared to the control group following the end of the program. We had the opportunity to test this hypothesis by using heart rate data collected during the RCT carried out at the MMFA. This study aims to compare changes in heart rate among older adults participating in the MMFA-based art activity and their control counterparts over the 3 month-period of the study.

## 2 Materials and methods

### 2.1 Population

**Figure 1:**
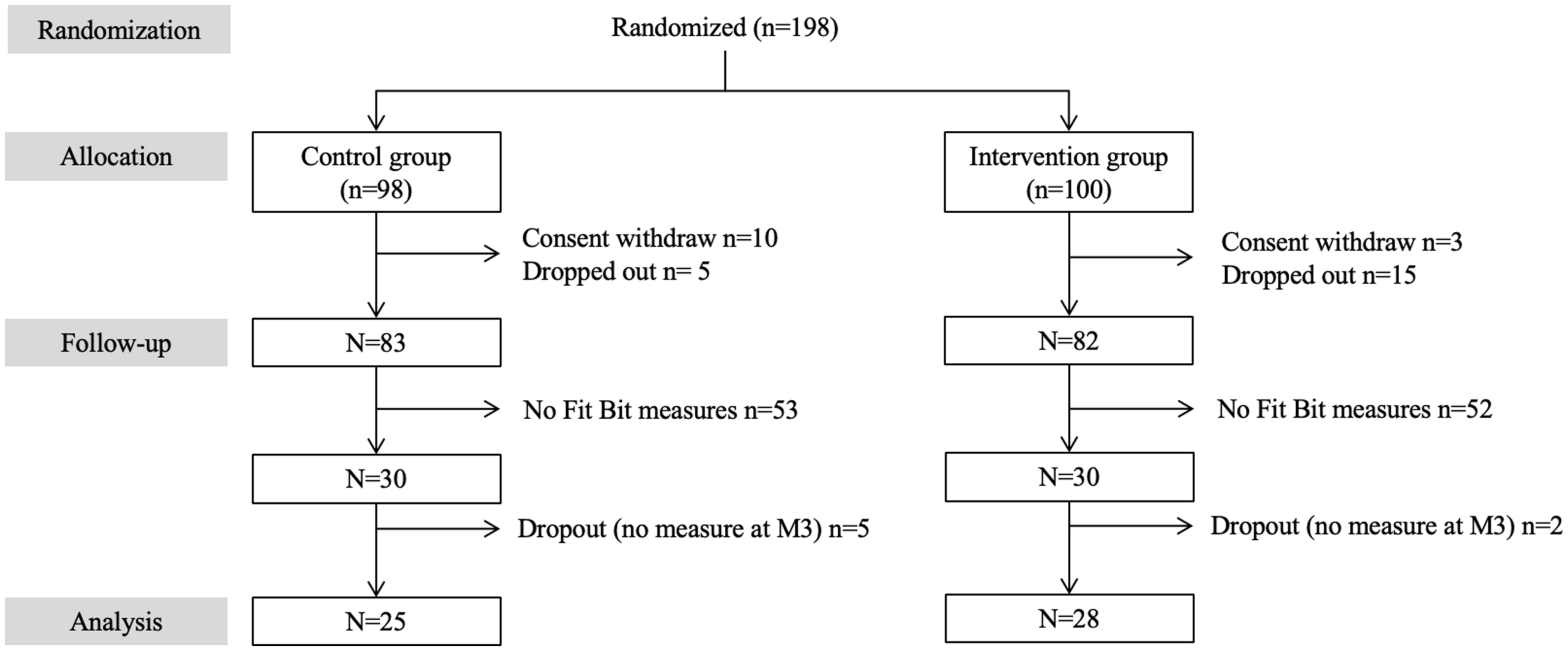
Consort flow diagram detailing selection and follow-up of participants in the RCT.

A total of 198 individuals living in the urban area of Montreal (Quebec, Canada) were enrolled in the RCT and randomized to intervention (*n* = 100) and control groups (*n* = 98) between March 2019 and November 2019. Recruitment, assessment, and follow-up have been previously reported (8). To summarize, participants were MMFA visitors aged 65 years and over who lived at home or in an unassisted residence. Among the 198 enrolled participants, 3 (1.5%) withdrew their consent in the intervention group and 10 (5.1%) in the control group before the baseline assessment. In addition, 20 (10.1%) participants dropped out during the 3-month follow-up period (15 in the intervention group and 5 in the control group). Among the 165 participants who completed the 3-month period of assessment, 60 (30.3%) participants agreed to record their heart rate: 30 in the intervention group and 30 in the control group. Seven (11.7%) participants were excluded from the present study because of technical issues with heart rate recording. As a result, 53 (26.8%; mean age 71.0 ± 5.1; 84.9% female) had their heart rate recorded and were selected for the present study (28 participants in the intervention group and 25 in the control group). A flow diagram detailing participant selection and follow-up in the RCT is presented in the Figure 1.

### 2.2 Study design

The design was a RCT in two parallel groups (intervention vs. control). Block randomization with a block size of 1 was used to divide the groups. Participant in the intervention group were not blinded but investors were blinded to the intervention and the results of assessments. The CONSORT guidelines were applied for this RCT (22). The project number on the ClinicalTrials.gov website is NCT03679715.

### 2.3 Intervention

The intervention consisted of weekly participatory art activities carried out at the MMFA over a 3-month period. These activities have previously been described in detail (8). Briefly, 2-h workshops were carried out in a group setting over the course of 12 consecutive weeks. Participants were divided into two groups, meeting either on Tuesdays or Wednesdays. Each workshop was conducted by two arts and culture facilitators who led interactive arts and crafts activities targeting creativity (e.g., abstract painting/life drawing), craft skills (e.g., bookbinding/mini-fanzine) or fine motor skills (e.g., rolled paper and stained-glass painting). The workshops were structured as follows: (i) presentation of the activity’s objective, (ii) choice of medium, (iii) technical instruction.

### 2.4 Assessment and outcomes

Clinical information was collected before the first workshop at baseline (M0) and after the last workshop at the end of the 3-month period (M3) and comprised age, sex, and the Centre of Excellence Self-AdMinistered questionnaire (CESAM) (23). The CESAM is a validated questionnaire that assesses different subdomains of health via 20 close-ended questions, including polypharmacy (i.e., number of therapeutic classes daily taken ≥5), activities of daily living and instrumental activities of daily living, mood and history of falls in the past 12 months. CESAM was filled out by participants under the supervision of Principal Investigator representatives at their place of living. The score ranges from 0 (i.e., best health condition) to 18 (i.e., worst health condition).

Heart rate was recorded with a Fitbit Alta HR, which is a watch-like device that continuously tracks HR using photoplethysmography (11). Heart rate was me measured twice in this RCT. The first recording was performed over the 2 days before the first workshop (M0) and the second recording was performed over the 2 days after the last workshop (M3). The choice of 2 days of recording has been made with the objective to obtain sufficient data for statistical analysis. The Fitbit Alta HR smartwatch was selected because of its simple setup and usability (11, 18–20). Participants were instructed to wear the device on the wrist of their choice and to remove it only in situations where it may get wet (e.g., doing the dishes, showering) over 5 consecutive days. They were also instructed not to change wrists. For the present study, we used the mean heart rate per hour during active hours (i.e., between 12 pm and 6 pm), inactive hours (i.e., between 12 am and 6 am), and over the course of a full day (i.e., 24 h). We calculated the change of heart rate parameters during the first (M0) and second assessments (M3) using the following formula: [(M3 value − M0 value)/((M3 value + M0 value)/2) × 100].

### 2.5 Ethical considerations

The Jewish General Hospital Ethics Committee (Montreal, Quebec, Canada) approved the present study (2019-1493). All study participants provided their written informed.

### 2.6 Statistics

Participants’ characteristics were described using means, standard deviations (SD), frequencies and percentages. Inter and intra-group comparisons were performed with Mann–Whitney *t-*tests, Wilcoxon tests, Chi-squared or Fisher’s Exact tests, as appropriate. Linear regressions were used to examine the association between changes in heart rhythm parameters between M0 and M3 (used as dependent variable, with a separate model for each heart rate parameter) and MMFA-based art activity (used as independent variable), adjusted for participants’ baseline characteristics. The fixed threshold of significance for *p*-values was <0.05.

## 3 Results

**Figure 2:**
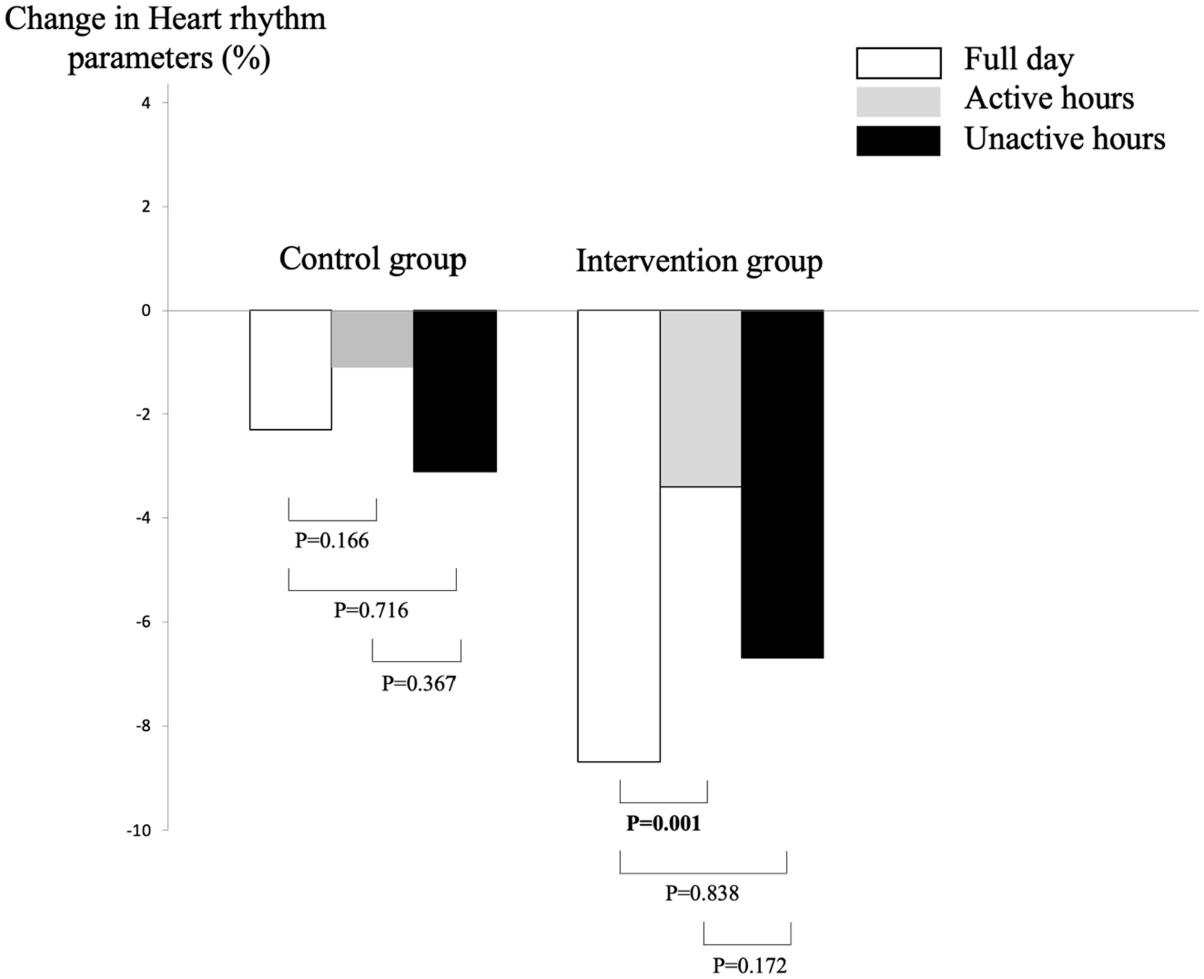
Comparison of changes in heart rate parameters between baseline and the end of the museum-based art activity at 3 months in the control (*n* = 25) and intervention groups (*n* = 28). Comparisons based on Wilcoxon test. Change calculated from the formula: [(M3 value – M0 value)/(M3 value + M0 value)/2) × 100]. *p*-value significance (i.e., <0.05) indicated in bold.

No significant difference for clinical characteristics and heart rate parameters was found between the intervention and the control groups at baseline (Table 1). At M3, mean heart rate for full day (70.2 ± 6.9 vs. 74.3 ± 6.5 with *p* = 0.018) and active hours (78.4 ± 8.1 vs. 82.7 ± 8.9 with *p* = 0.028) were significantly slower in the intervention group compared to the control group. There was no significant difference between groups for inactive mean heart rate (59.9 ± 6.4 for the intervention group vs. 63.2 ± 7.0 for the control group with *p* = 0.028). The decrease in full day mean heart rate observed between M0 and M3 in the intervention group was higher than the control group (−8.1 ± 7.1% vs. −2.3 ± 8.4% with *p* = 0.030 for full day). There was no significant difference between groups for change in active hours (−3.4 ± 8.4% in the intervention group vs. −1.1 ± 9.5% in the control group with *p* = 0.310) and in inactive hours (−6.7 ± 10.7% in the intervention group vs. −3.1 ± 9.3% in the control group with *p* = 0.061). Comparison of changes in heart rate parameters between M0 and M3 showed that the decrease in heart rate was greater for full hours compared to active hours in the intervention group (*p* = 0.001; Figure 2). Multiple linear regressions showed that decrease in heart rate between M0 and M3 for full day heart rate was negatively associated with MMFA-based art activity (coefficient of regression beta = −6.2 with *p* = 0.010; Table 2).

**Table 1:** Characteristics of participants (*n* = 53).

	Participants		*p*-value*
	Control (*n* = 25)	Intervention (*n* = 28)	
**Baseline characteristics**			
Age > 80, *n* (%)	2 (8.0)	3 (10.7)	0.736
Male, *n* (%)	2 (8.0)	6 (21.4)	0.173
Number of drugs daily taken ≥5, *n* (%)	20 (80.0)	23 (82.1)	0.842
Abnormal ADL score (/6)^†^, *n* (%)	4 (16.0)	2 (7.1)	0.310
Abnormal IADL score (/4)^‡^, *n* (%)	2 (80.0)	0	0.127
Bad mood^||^, *n* (%)	7 (28.0)	6 (21.4)	0.579
History of falls in the past 6 months, *n* (%)	10 (40.0)	8 (28.6)	0.380
CESAM frailty score (/18)^§^, mean ± SD	3.1 ± 2.2	2.5 ± 2.5	0.233
**Heart rhythm parameters (per hour), mean ± SD**			
*Full day*			
M0	76.1 ± 7.7	76.1 ± 7.9	0.831
M3	74.3 ± 6.5	70.2 ± 6.9	**0.018**
Variation between M0 and M3 (%)^¶^	−2.3 ± 8.4	−8.1 ± 7.1	**0.030**
*Active hours^#^*			
M0	83.6 ± 9.9	81.1 ± 9.1	0.581
M3	82.7 ± 8.9	78.4 ± 8.1	**0.028**
Variation between M0 and M3 (%)^¶^	−1.1 ± 9.5	−3.4 ± 8.4	0.310
Inactive hours^**^			
M0	65.3 ± 7.5	64.0 ± 6.0	0.378
M3	63.2 ± 7.0	59.9 ± 6.4	0.074
Variation between M0 and M3 (%)^¶^	−3.1 ± 9.3	−6.7 ± 10.7	0.061

SD, standard deviation; ADL, activities of daily living with score ranges between 0 (dependent) and 6 (independent); IADL, instrumental activities of daily living with score ranges between 0 (non-autonomous) and 4 (autonomous); M0, baseline, M3, 3 months; CESAM, Centre of Excellence Self-AdMinistered Questionnaire.

^*^Comparison based on Mann–Whitney *t*-tests, Chi-squared or Fisher’s Exact tests, as appropriate; ^†^Score ≤ 5/6; ^‡^Score ≤ 3/4; ^||^Based on answer to the question “How do you feel today?” unhappy; ^§^Mean score calculated from computerized self-administered questionnaire composed of 20 questions providing a score ranging from 0 (vigorous) to 18 (severe frailty); ^¶^Calculated from the formula [((M3 value – M0 value)/((M3 value + M0 value)/2)) × 100]; ^#^Between 12 pm and 6 pm; ^**^Between 12 am and 6 am; *p*-value significance (i.e., <0.05) indicated in bold.

**Table 2:** Linear regressions showing the association between (a) changes in heart rhythm parameters between baseline and following the intervention (M3) (used as dependent variable, separated model for each heart rate parameter) and (b) museum-based art activity (used as independent variable) in participants (*n* = 53).

Change in heart rhythm parameters between M0 and M3^*^	β	[95% CI]	*p*-value
Full day	−6.2	[−10.8; −1.6]	**0.010**
Active hours^†^	−2.4	[−7.6; 2.8]	0.358
Inactive hours^‡^	−4.1	[−10.2; 1.9]	0.173

β, coefficient of regression beta; CI, confident interval; all models adjusted on baseline characteristics of participants (please see Table 1).

^*^Calculated from the formula [((M3 value – M0 value)/((M3 value + M0 value)/2)) × 100]; ^†^Between 12 pm and 6 pm; ^‡^Between 12 am and 6 am; *p*-value significance (i.e., <0.05) indicated in bold.

## 4 Discussion

The results show that the MMFA-based art activity significantly reduced full-day heart rate. Mixed results were shown for active hours and no effect was reported for inactive hours.

Mean full-day heart rate significantly decreased in the intervention group compared to the control group in our study. To the best of our knowledge, this is the first time that such an effect of museum-based art activity is being reported in older adults. The effect may be interpreted as a health benefit. Heart rate represents the balance between the sympathetic and parasympathetic nervous systems, with a slower heart rate indicating a better balance and thus a healthy state (15–18). This statement suggests that heart rate could be considered as proxy measure of healthy aging. The autonomic nervous system is essential for adapting to stressful events and its main function is to maintain homeostasis (15). Therefore, its maintenance and balance are essential for healthy aging (16). A slower full-day heart rate in older community dwellers suggests an improvement in the balance between the sympathetic and parasympathetic nervous systems, and thus a better health state. This result is consistent with the literature on the health effects of museum-based art activities for older adults (3–8). Indeed, previous studies have shown improvements in participants’ mental and physical health states (5–8). Because the physiological measure assessed in this study is a biomarker of physical health status, our results suggest an improvement in participants’ physical health through improved autonomic balance.

To date, however, museum-based art activities have been assessed using self-reported questionnaires (3–8). Thus, the reported change in full-day mean heart rate cannot be compared to the results of other studies. The decrease in mean full-day heart rate may be explained by an improvement in participants’ well-being, which influences the autonomic nervous system. Indeed, well-being contributes to stress regulation by limiting chronic system activations, thus improving overall health. Another explanation may be an increase in participants’ physical activity levels - they needed to walk to attend the museum activities - which is beneficial to the cardiac autonomic system. Thus, it is likely that their physical activity saw a significant increase during this study.

Regarding the active and inactive hours variables, mixed results for the former were demonstrated. The mean heart rate for active hours was lower in the intervention group compared to the control group at M3, but no significant change between M0 and M3 was observed. In addition, no effects of the intervention were observed for inactive hours. These non-conclusive results may be explained by the small number of participants, which leads to a lack of data to show significant effect. In addition, it may be suggested that the condition of inactive hours - which correspond to a resting condition - is not sensitive enough to observe an effect, because the heart rate is slower when compared to active hours.

The RCT design of the study is a major strength, as it provides the highest level of evidence on the effectiveness of an intervention. In addition, the use of a physiological biomarker bypasses the biases of self-administered questionnaires. However, there are certain limitations which should be considered. First, this RCT is a pilot study with a small sample size of participants, which may explain mixed and non-conclusive results for active and unactive hours, respectively. Second, recruiting the participants in the older visitors of museum may be a bias because were already interested in museum-based art activities and, thus, may expose to show no or limited effects of the intervention. In addition, because of participants interest in art, the benefices in art-based activities may be limited to older people who are interested in art. Third, we used mean heart rate values, whereas heart rate variability measurements would provide more insight into autonomic nervous system processes. Fourth, we did not have access to the patients’ treatments and, therefore, could not consider the effects of medications like beta-blockers on heart rate. However, the participants of control and intervention groups were similar. Thus, it may be suggested that if some of them took heart rate lowering medications, the proportion in each group was the same that limited the effect on the results.

In conclusion, MMFA-based art activities were associated with a significant decrease in full-day mean heart rate, suggesting a health benefit in older community dwellers who participated in the RCT. It would be interesting to reproduce this study with a larger number of participants and to measure heart variability, in order to confirm the health benefits observed in this study. The perspective of confirmation of heart rate benefits of museum-based art activities in an aging population may be useful for promotion of healthy aging and prevention of adverse effects of stress on mental and physical health.

## Data availability statement

The raw data supporting the conclusions of this article will be made available by the authors, without undue reservation.

## Ethics statement

The studies involving humans were approved by The Jewish General Hospital Ethics Committee (Montreal, Quebec, Canada). The studies were conducted in accordance with the local legislation and institutional requirements. The participants provided their written informed consent to participate in this study. Written informed consent was obtained from the individual(s) for the publication of any potentially identifiable images or data included in this article.

## Author contributions

OB conceived of and designed the experiments, performed the experiments, and contributed reagents, materials, analysis tools or data. MC and OB analyzed and interpreted the data and writing of the manuscript. JM, AP, and AG revision of manuscript. All authors contributed to the article and approved the submitted version.
